# Induction of IL-12 Production in Human Peripheral Monocytes by *Trypanosoma cruzi* Is Mediated by Glycosylphosphatidylinositol-Anchored Mucin-Like Glycoproteins and Potentiated by IFN-***γ*** and CD40-CD40L Interactions

**DOI:** 10.1155/2014/345659

**Published:** 2014-07-09

**Authors:** Lúcia Cristina Jamli Abel, Ludmila Rodrigues Pinto Ferreira, Isabela Cunha Navarro, Monique Andrade Baron, Jorge Kalil, Ricardo Tostes Gazzinelli, Luiz Vicente Rizzo, Edecio Cunha-Neto

**Affiliations:** ^1^Laboratory of Immunology, Heart Institute (InCor), School of Medicine, University of São Paulo, 05403-001 São Paulo, SP, Brazil; ^2^Division of Clinical Immunology and Allergy, School of Medicine, University of São Paulo, 01246-000 São Paulo, SP, Brazil; ^3^Institute for Investigation in Immunology (III), INCT, 05403-001 São Paulo, SP, Brazil; ^4^Research Center René Rachou, Oswaldo Cruz Foundation (FIOCRUZ), 30190-002 Belo Horizonte, MG, Brazil; ^5^Laboratory of Immunopathology, Department of Biochemistry and Immunology, Institute of Biological Sciences, Federal University of Minas Gerais, 31270-910 Belo Horizonte, MG, Brazil; ^6^Division of Infectious Diseases and Immunology, Department of Medicine, University of Massachusetts Medical School, Worcester, MA 01605, USA; ^7^Hospital Israelita Albert Einstein, Avenida Albert Einstein 627-701, 2 Subsolo Bloco A., 05651-901 São Paulo, SP, Brazil

## Abstract

Chagas disease, caused by the protozoan parasite *Trypanosoma cruzi* (*T. cruzi*), is characterized by immunopathology driven by IFN-*γ* secreting Th1-like T cells. *T. cruzi* has a thick coat of mucin-like glycoproteins covering its surface, which plays an important role in parasite invasion and host immunomodulation. It has been extensively described that *T. cruzi* or its products—like GPI anchors isolated from GPI-anchored mucins from the trypomastigote life cycle stage (tGPI-mucins)—are potent inducers of proinflammatory responses (i.e., cytokines and NO production) by IFN-*γ* primed murine macrophages. However, little is known about whether *T. cruzi* or GPI-mucins exert a similar action in human cells. We therefore decided to further investigate the *in vitro* cytokine production profile from human mononuclear cells from uninfected donors exposed to *T. cruzi* as well as tGPI-mucins. We observed that both living *T. cruzi* trypomastigotes and tGPI-mucins are potent inducers of IL-12 by human peripheral blood monocytes and this effect depends on CD40-CD40L interaction and IFN-*γ*. Our findings suggest that the polarized T1-type cytokine profile seen in *T. cruzi* infected patients might be a long-term effect of IL-12 production induced by lifelong exposure to *T. cruzi* tGPI-mucins.

## 1. Introduction

The protozoan parasite* Trypanosoma cruzi* is the causative agent of Chagas disease, which affects approximately 15 million people in South and Central America [1, 2]. It is estimated that about 30% of infected individuals will develop severe chronic forms of the disease, especially the often fatal Chagas disease cardiomyopathy (CCC) [1–4]. Intracellular protozoan parasites are potent stimulators of innate and cell-mediated immunity. The induction of macrophage proinflammatory cytokines by ligands of innate immunity receptors of* T. cruzi* is considered important in the control of infection and outcome of Chagas disease [5, 6]. It has been extensively described that glycosylphosphatidylinositol-anchored mucins-like glycoproteins from* Trypanosoma cruzi* trypomastigotes (tGPI-mucins) activate murine macrophages* in vitro* to produce the proinflammatory cytokines tumor necrosis factor *α* (TNF-*α*) and interleukin- (IL-) 12 as well as nitric oxide (NO) [7, 8]. The bulk of evidence establishes that IL-12 and IL-12 driven Th1 cytokines, the ones involved in delayed-type hypersensitivity, are induced during acute infection with* T. cruzi* in mice, playing an obligatory role in parasite clearance and host survival [9–12].* T. cruzi* tGPI-mucins were shown to initiate the inflammatory response through an activation of Toll-like receptors TLR2 [7, 13]. Different components from this parasite are capable of activating TLRs in dendritic cells and macrophages, like the unmethylated CpG motifs present in* T. cruzi* genome, were identified as a TLR9 agonist [14].* T. cruzi* chronically infected Chagas disease patients display a Th1 cytokine profile [15] which is even more pronounced among CCC patients [16, 17]. It has been described that certain infectious agents, like* Mycobacterium tuberculosis*, possess molecules stimulating innate immunity that can shift the systemic cytokine environment and modify clinical immune profiles [18]. Our group and others have previously reported that heart-infiltrating T cells predominantly produce IFN-*γ* and TNF-*α*, suggesting that such Th1 T cells play an important pathogenetic role in heart tissue damage in CCC [16, 19–22]. Even though acute* T. cruzi* infection induces IL-12 production in mice, little is known about whether* T. cruzi* or tGPI-mucins exert a similar action in humans. We have previously described the isolation of live* T. cruzi* trypomastigotes outgrowing from a heart biopsy fragment from a CCC patient [23], routinely cultured for the study of outgrowing heart-infiltrating T cells [16, 24]. In order to study whether* T. cruzi* and tGPI-mucins could directly induce the production of the Th1-inducing cytokine IL-12 in human cells, we studied the cytokine profile in naturally infected supernatants of heart-infiltrating mononuclear cells. We also assessed the effect of cocultivation of* T. cruzi* and tGPI-mucins with peripheral blood mononuclear cells and purified monocytes on IL-12 production. Finally, we assessed the role of IFN-*γ* and CD40L signaling on* T. cruzi* and tGPI mucin-induced IL-12 production.

## 2. Methods

### 2.1. Parasites

The Y strain of* T. cruzi* was maintained in fibroblast cultures and was used as parasite source for purification of tGPI-mucins. For the trypomastigote culture, L-929 fibroblasts were initially infected with blood trypomastigotes in a ratio of one parasite per cell. The tissue culture trypomastigotes were continuously passed in L-929 fibroblast cultures. The infected cultures were maintained in Dulbecco's modified Eagle's medium (DMEM) containing 5% fetal calf serum (FCS) at 33°C in 5% CO_2_. After 4 or 5 days of culture, the parasites were collected daily and centrifuged at 40 g at 4°C for 10 min for cellular debris separation, followed by another centrifugation at 700 g at 4°C for 10 min. The resulting pellet containing live trypomastigotes was used to purify GPI-mucins.

### 2.2. Purification of tGPI-Mucins

The GPI-mucins were isolated from* T. cruzi* trypomastigotes as described previously [7, 8] using sequential organic extraction followed by hydrophobic-interaction chromatography in an Octyl-Sepharose column (Amersham Pharmacia Biotech, Uppsala, Sweden) and elution with a propan-1-ol gradient (5–60%).

### 2.3. Heart-Infiltrating T Cell Lines

T cell lines were established from endomyocardial biopsy explants from CCC patients as described [16]. Briefly, biopsy tissue was minced and seeded on to 96-well flat bottom plates in the presence of IL-2 and irradiated peripheral blood mononuclear cells (PBMC) until lymphoblast outgrowth was observed; T cell lines were expanded by restimulation every two weeks with 5 *μ*g phytohemagglutinin (PHA) and irradiated PBMC. PBMC were obtained from blood of healthy donors and separated by density gradient centrifugation with Ficoll-Hypaque^*R*^. All cells were cultured in Dulbecco's modified Eagle's medium supplemented with 2 mM L-glutamine, 1 mM sodium pyruvate, MEM's nonessential amino acids and MEM's vitamins (all from GIBCO, Grand Island, NY, USA), 50 *μ*g/mL gentamicin, 10 mM HEPES buffer, and 10% normal human serum (complete medium). This protocol has been approved by the Institutional Review Board of the University of São Paulo School of Medicine and all subjects provided informed consent.

### 2.4. *T. cruzi* Coculture/GPI Treatment

Ten to 12 days after the last PHA stimulation, heart-infiltrating T cell lines (from four different individuals, in separate experiments) were stimulated in the presence of irradiated PBMC (5 × 10^5^/well) plus 5 *μ*g/mL PHA and supernatants were obtained after 48 h incubation. In another set of experiments, culture conditions included variable components: irradiated PBMC, heart-infiltrating T cell lines (from four different individuals), 5 *μ*g/mL PHA, 5 × 10^4^ Y strain* T. cruzi* trypomastigotes obtained from LLC-MK2 monolayer cell culture, or 10 pmol/mL of* T. cruzi* tGPI-mucins. Blocking/neutralizing monoclonal antibodies against CD28, CD40, and IFN-*γ* (Pharmingen, La Jolla, CA) were employed in selected experiments.

### 2.5. Human Monocytes

Human monocytes were obtained by leukapheresis of normal volunteers at the blood bank of National Institutes of Health (Bethesda, MD). After density sedimentation of the mononuclear cells with lymphocyte separation medium (Organon, Teknika, Durham, NC), the monocytes were purified by counterflow centrifugal elutriation, as described previously [25], except that pyrogen-free PBS was used in the elutriation procedure. Monocytes were enriched >90% as determined by morphology, non-specific esterase staining, and flow cytometry. The purification procedure did not activate the monocytes, as shown by the fact that, after overnight incubation at 37°C in suspension, 4% of the cells were IL-12R positive or spontaneously secreted any of the cytokines measured. After purification, monocytes were left at 4°C overnight and then transferred to 5 mL polystyrene Falcon tubes (Becton Dickson Labware, Lincoln Park, NJ) and cultured for 24 h in the presence or absence of 10 pmol/mL tGPI-mucins, in the presence or absence of 100 units/mL of human IFN-*γ* (Genetech), as indicated. Culture supernatants were collected 48 h after stimulation for IL-12 determination.

### 2.6. Cytokine Measurements

Cytokines IFN-*γ*, IL-4, IL-2, IL-10, IL-12, and TNF-*α* were measured by double sandwich ELISA using the anti-human cytokine antibody pairs (R&D Systems, Minneapolis).

## 3. Statistical Analysis

Groups were compared by a nonparametrical test (Mann-Whitney Rank Sum Test) with GraphPad InStat software (version 5.0; GraphPad). Results were expressed as medians and interquartile ranges. *P* values were considered significant if <0.05.

## 4. Results

### 4.1. *T. cruzi* Outgrowth from Endomyocardial Biopsies from CCC Patients Induces the Production of T1-Type Cytokine Profile

**Figure 1:**
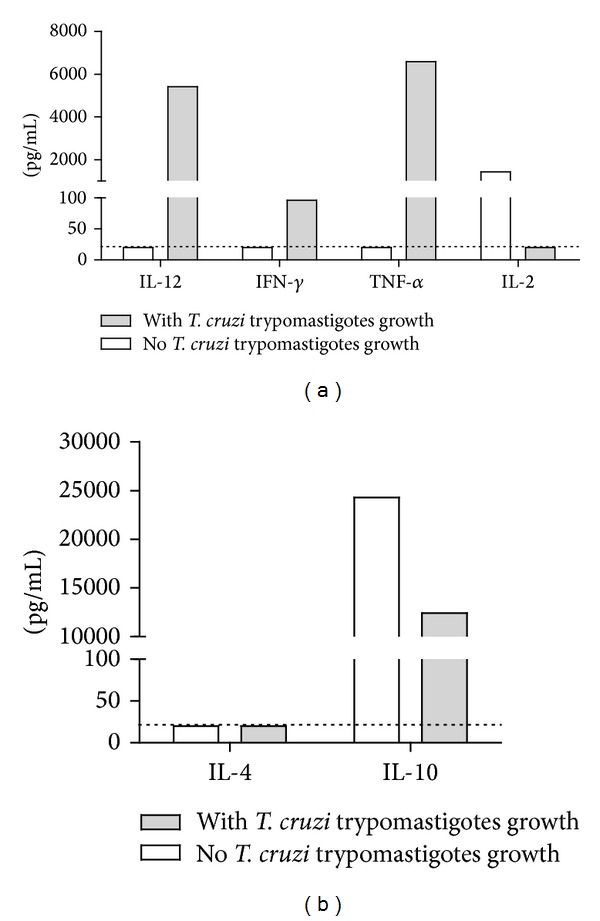
*T. cruzi* trypomastigotes outgrowth from endomyocardial biopsies from CCC patients induces the production of T1-type cytokine profile.

We routinely cultured T cell lines from endomyocardial biopsies from CCC patients for the isolation of T cell lines. In one of these biopsy explants, highly motile* T. cruzi* trypomastigotes were observed in some of the seeded wells, indicating that the tissue fragments in those wells probably contained a* T. cruzi* pseudocyst. We therefore compared PHA-stimulated cytokine production in the supernatant from the T cell line established from wells containing live* T. cruzi* parasites (with* T. cruzi* trypomastigote growth) with the cell line derived from wells of the same biopsy devoid of* T. cruzi* (no* T. cruzi* trypomastigote growth). Figures 1(a) and 1(b) depict the cytokine profile of the T cell line obtained from the* T. cruzi*-positive wells as compared to the T cell line of the same sample, obtained from wells where no* T. cruzi* trypomastigotes were observed. As can be seen,* T. cruzi* trypomastigotes outgrowth induced the production of IL-12, TNF-*α*, and IFN-*γ*, with undetectable levels of IL4. The presence of* T. cruzi* strongly reduced the levels of IL-2 and mildly reduced IL-10 levels.

### 4.2. *T. cruzi* Trypomastigotes Induce IL-12 Production by Human PBMC, Which Is Potentiated by Activated T Cells

**Figure 2:**
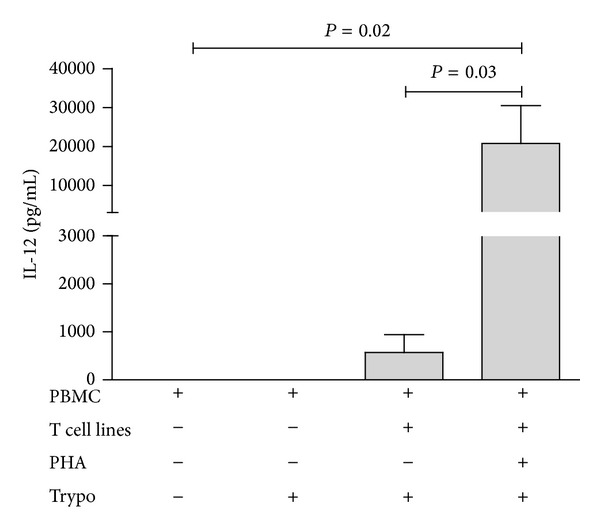
*T. cruzi*-induced IL-12 production is potentiated by activated T cells. Results come from 4 distinct experiments. Groups were compared by a nonparametrical test (Mann-Whitney Rank Sum Test) with GraphPad InStat software (version 5.0; GraphPad). Results were expressed as medians and interquartile ranges. *P* values were considered significant if <0.05.

To further investigate the phenomenon observed in the endomyocardial biopsies wells we assayed cytokine production in supernatants of human PBMC in the presence of living* T. cruzi* and/or PHA-activated T cells. As shown in Figure 2,* T. cruzi* trypomastigotes can induce moderate production of IL-12 directly on irradiated PBMC or in cocultures of PBMC and T cells. However, coculture with PHA-activated T cell lines induced a 10- to 100-fold increase in IL-12 production by irradiated PBMC.

### 4.3. GPI-Mucins from* T. cruzi* Trypomastigotes Induce IL-12 Production by Human Monocytes

**Figure 3:**
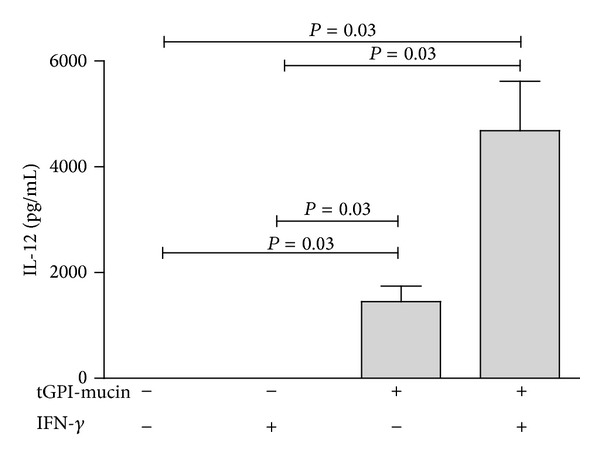
tGPI-mucins from* T. cruzi* induce IL-12 production by human monocytes. Results come from 3 distinct experiments. Groups were compared by a nonparametrical test (Mann-Whitney Rank Sum Test) with GraphPad InStat software (version 5.0; GraphPad). Results were expressed as medians and interquartile ranges. *P* values were considered significant if <0.05.

We also tested if purified tGPI-mucins could activate isolated PBMC-derived monocytes* in vitro* to produce IL-12. As shown in Figure 3, tGPI-mucins induce significant production of IL-12 by human monocytes, which is further potentiated after IFN*γ* priming of cells.

### 4.4. Induction of IL-12 Production by* T. cruzi* or tGPI-Mucins Is Dependent on IFN-*γ* and CD40-CD40L Interactions

**Figure 4:**
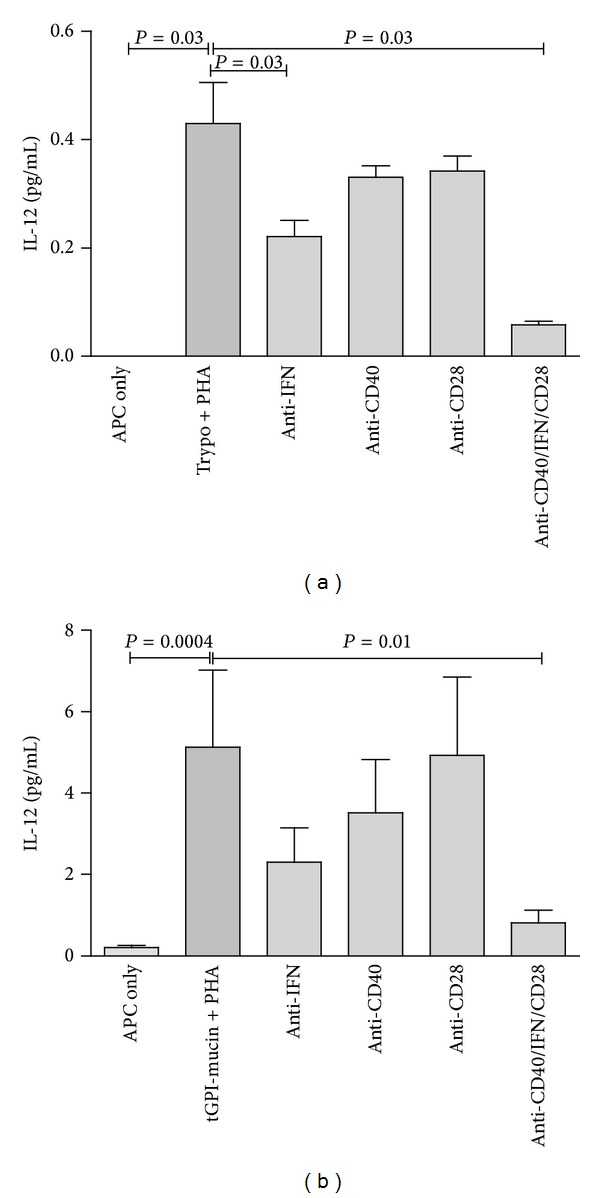
Potentiation of IL-12 production induced by (a) live* T. cruzi* trypomastigotes or (b) tGPI-mucins is dependent on IFN-*γ* and CD40. Irradiated PBMC were incubated with T cell lines, PHA (5 ug/mL), and different blocking antibodies (anti-IFN-*γ*, anti-CD40, anti-CD28, and anti-IFN/CD40/CD28). Results come from 4 distinct experiments. Groups were compared by a nonparametrical test (Mann-Whitney Rank Sum Test) with GraphPad InStat software (version 5.0; GraphPad). Results were expressed as medians and interquartile ranges. *P* values were considered significant if <0.05. Significance bars are shown in comparison with trypo + PHA or tGPI-mucin + PHA.

In an attempt to study the mechanisms underlying* T. cruzi*-induced potentiation of IL-12 production by human monocytes, we cocultured these cells with PHA-activated T cell lines, 5 × 10^4^ *T. cruzi* Y strain living trypomastigotes, or tGPI-mucins and added neutralizing/blocking antibodies to human IFN-*γ*, CD40, and CD28. Results indicated that blocking IFN-*γ* or CD40 individually caused approximately 50% and 35% of inhibition of IL-12 production, respectively, while anti-CD28 showed negligible inhibition. The combined effect of the three antibodies induced 85% of inhibition, suggesting that most of the IL-12-inducing effects of PHA-activated T cell lines are due to IFN-*γ* production and CD40-CD40L interactions (Figure 4(a)). Similar results were obtained when tGPI mucin was used as stimulus (Figure 4(b)) suggesting that these molecules may be the effectors in the* T. cruzi*-induced IL-12 production in humans, as has been previously described in mice [8].

## 5. Discussion

In this paper, we observed that both living trypomastigotes and tGPI-mucins are potent inducers of IL-12 production in human monocytes and that this effect depends on CD40-CD40L interaction and IFN-*γ* signaling. The finding that spontaneous outgrowth of parasites in culture cells derived from chronically infected myocardium induced the production of T1-type proinflammatory cytokines, like IL-12, TNF*α*, and IFN*γ*, is in accordance with data from murine models from other studies [26–30]. These cytokines, which are induced during acute infection with* T. cruzi* in mice, play an obligatory role in parasite clearance and host survival [26, 29]. However, the same immunological pattern may participate in mechanisms of tissue damage in Chagas disease, indicating that protective and pathological responses must share important characteristics in this context [31]. When parasites were deliberately added to cocultures of irradiated PBMC and activated T cell lines, we observed again high levels of IL-12 expression in PBMC, although the* T. cruzi* stimulus itself was capable of inducing some IL-12 expression by PBMC in the absence of activated T cells. This corroborates the findings obtained with cultures with spontaneous outgrowth of* T. cruzi* trypomastigotes, where we had PHA-activated cell lines and* T. cruzi* trypomastigotes. Our results indicate that PBMC-derived monocytes are the cell population responding to tGPI-mucins with* in vitro* IL-12 production. Although we already observed induction of IL-12 production by monocytes using tGPI-mucins as a first signal (microbial stimulus via TLR-2), maximal levels of IL-12 are reached only after a second signal through the presence of IFN-*γ*, as it has been reported by other studies [32, 33]. The alkylacylglycerolipid component of tGPI-mucins [7] is capable of triggering Toll-like receptors-2 at subnanomolar concentrations [13]. Moreover, macrophages derived from Tlr2^−/−^ or Myd88^−/−^ mice are less responsive to tGPI-mucins, further confirming the possible role of the TLR pathway in this process [34]. Our findings that anti-IFN-*γ* and anti-CD40L neutralizing antibodies were able to significantly reduce IL-12 production indicate this phenomenon is mediated by IFN-*γ* and CD40-CD40L interactions. This can be explained by the fact that, in this context, T cells are likely to be the major source of IFN-*γ* and membrane CD40L, activators of macrophages involved in many aspects of parasite control [11, 35]. As previously described by Chaussabel et al. CD40 ligation in* T. cruzi-infected* mice has a protective effect because it is related to upregulation of IL-12 as well as NO by a direct stimulation of INF-*γ* activated macrophages [36]. Previous studies also showed that the CD40-CD40L signaling pathway mediated protective effect with other pathogens such as* Leishmania* [37],* Schistosoma mansoni* [38],* Cryptococcus neoformans* [39],* Cryptosporidium parvum* [40], and* Pneumocystis carinii* [41]. The enhanced production of IFN-*γ*, TNF-*α*, and nitric oxide associated with CD40/CD40L signaling is thought to be responsible for this protective effect. It was shown that IFN*γ* stimulus also upregulates the transcription factor T-bet [42], which in turn maintains IL-12R*β* chain expression [43], possibly resulting in a positive feedback loop that, consequently, keeps the shift towards a Th1 response in Chagas disease. In summary, our data suggest that the T1-type cytokine profile found in the peripheral blood and among heart-infiltrating T cells is related to previous or ongoing encounters with IL-12 generated as a response to* T. cruzi* GPI-anchored mucin-like glycoproteins.
